# Community Health Workers as Allies in Hypertension Self-Management and Medication Adherence in the United States, 2014

**DOI:** 10.5888/pcd13.160236

**Published:** 2016-12-29

**Authors:** Caitlin G. Allen, J. Nell Brownstein, Anamika Satsangi, Cam Escoffery

**Affiliations:** 1 Emory University, Rollins School of Public Health, Atlanta, Georgia. Ms Allen is now affiliated with Boston University, Department of Medicine, Boston, Massachusetts; 2 Emory University, Rollins School of Public Health, Atlanta, Georgia.

## Abstract

**Introduction:**

Rates of hypertension control remain low among underserved populations in the United States; moreover, disparities in hypertension-related cardiovascular disease death are increasing. Community health workers (CHWs) can address barriers to hypertension control among underrepresented and diverse populations. We identify unique roles CHWs play in hypertension self-management and medication adherence.

**Methods:**

In 2014, we conducted a mixed methods study with an online survey of 265 CHWs and 23 telephone interviews. The survey and interview guide contained questions about CHWs’ roles in hypertension self-management and hypertension medication adherence. We used descriptive statistics to analyze survey data and used inductive thematic analysis for the qualitative data.

**Results:**

CHWs described working in partnership with patients and various health care providers to assist people in hypertension self-management. Roles were flexible and multifaceted but patient-driven. CHWs used various delivery methods to assist patients in overcoming barriers to medication adherence. CHWs interacted with patients primarily through individual clinical sessions or home visits. On average, they visit about 8 times per month, about 40 minutes per visit, over 7 months. CHWs often addressed barriers related to medicine-taking and refills and support patient–provider communications.

**Conclusion:**

Results from this study will help health care professionals, policy makers, and academics better understand the work of CHWs. CHWs are important provider allies for improving hypertension prevention and self-management, especially among underserved and diverse populations in the United States.

## Introduction

Approximately 30% of adults in the United States have hypertension, which places them at a greater risk for heart disease and stroke, the first and fifth leading causes of death (1,2). Rates of hypertension control remain low among underserved populations.

Underserved populations have barriers that limit their health, including uninformed health beliefs and knowledge, diverse values, insufficient access to culturally appropriate screening and treatment, absence of disease self-management skills, and limited access to resources (eg, healthy foods and opportunities for physical activity) (3,4). Medication adherence is an important but underemphasized facet of hypertension self-management and treatment (5). Rates of medication nonadherence are high among low-income adults with multiple chronic diseases or lack of prescription drug coverage (6,7).

Although medical office visits are opportunities for patients to be educated about medication adherence and self-management (8–10), various challenges deter providers from delivering essential information about prescriptions and lifestyle modifications to their patients (11). Infrequent provider interactions are insufficient to sustain hypertension self-management (HSM) behavior changes; long-term lifestyle skills and support are required (12).

Community health workers (CHWs) are nonclinical, frontline public health workers who are trusted members of the community and who facilitate access to services and improve quality and cultural competence of service delivered (13). Strong evidence supports the effectiveness of CHWs as interventionists, in a team-based care model and in the community, for improved blood pressure control and management (14).

Our study explores the mechanisms by which CHWs produce results; it presents CHWs’ perspectives about their roles in HSM and medication adherence by answering 2 questions: 1) What are CHWs’ roles in HSM? and 2) How do CHWs support hypertension medication adherence?

## Methods

We conducted a mixed-methods study with an online survey of 265 CHWs and 23 telephone interviews from August to October 2014. All aspects of this study were approved by the Emory University Institutional Review Board.

The study population for this project was US CHWs nationwide. We used a sampling frame of CHWs on listserves affiliated with American Public Health Association–sponsored CHW networks and associations representing 19 states. The inclusion criteria were people aged 18 or older who considered themselves CHWs and could speak English. In total, 434 people visited the survey link; of those, 369 (85%) were CHWs and 265 (61%) consented to participate. The survey was open from August 21 to October 17, 2014, with reminders sent every other week through the listserve. At the end of the survey, participants were asked if they were interested in participating in an interview. Participants who self-selected to be interviewed (n = 64) were placed into a pool of potential interviewees and then purposefully selected for in-depth semistructured interviews conducted via telephone. Selection was based on whether the participant responded to more than 50% of the survey and responded to the hypertension section of the survey.

Both the quantitative and qualitative measurements aimed to answer the proposed research questions. The survey had 56 questions (multiple choice responses, free response, and Likert scale responses) and was conducted online using SurveyMonkey. The survey instruments explored roles in hypertension management, training competencies for delivering HSM education, and hypertension medication adherence. In addition, personal and organizational characteristics were assessed. We framed hypertension management questions around the 5 dimensions of adherence named by the World Health Organization (WHO) (23): health system and health care team factors, social and economic factors, condition-related factors, therapy-related factors, and patient-related factors. The qualitative aspects of this study were designed to understand ways CHWs support patients with hypertension: 21 open-ended questions with probes were used. All questions were at less than an 8th-grade reading level based on the Flesch-Kincaid readability test. The instruments were designed based on literature review and derived from various documents (4,5,15–17). Content experts and 4 CHW consultants reviewed both instruments for content validity and relevance. The telephone interviews lasted an average of 53.5 minutes.

Survey data were downloaded into SPSS version 22 (IBM Corporation) and Excel (Microsoft, Inc) for data analysis. We conducted descriptive analysis for CHWs’ personal and organizational characteristics and roles in management of chronic disease, hypertension, and medication adherence. Interviews were recorded and transcribed verbatim, quality controlled by the interview team, and coded for major themes based on the research question. We used inductive thematic analysis for qualitative analysis (18). A codebook was developed and adapted upon completion of a consensus review between 2 primary coders (C.G.A. and A.S.). Two coders separately coded the same 3 interviews and met for consensus and adoption of the codebook. The codes were compiled in MaxQDA version 11 (VERBI GmbH) and major codes were retrieved individually.

## Results

CHWs who responded to the survey (n = 160) were mostly female (88.3%) and had a high school diploma, general equivalency diploma, or higher; 45.4% (n = 69) were Hispanic/Latino, 25.7% black or African American, and 25.0% non-Hispanic white. They had a mean (standard deviation [SD]) age of 43.1 (12.8) years.

### CHWs as partners in hypertension self-management

The most frequently cited roles by CHWs who responded to questions about HSM (n = 141) included education on healthy diet (83.7%) or low-sodium diet (79.4%), helping patients understand that they should not stop taking their medicine without talking to their doctor (73.8%), and assisting patients with keeping doctor’s appointments (73.1%). CHWs described their roles as flexible and multifaceted but patient-driven (Table 1). CHWs described working in partnership with patients and providers to assist people in HSM. As one CHW explained: “We don’t make any decisions for the patient, but rather help them with goals and with just stay[ing] focused on what they have decided with the primary care physician” [Interview no. 1].

**Table 1:** Community Health Worker Roles in Hypertension Self-Management, United States, 2014 (n = 141)^a^

Role	No. (%)
Educate on healthy diet (rich in fruits and vegetables)	118 (83.7)
Educate on low-sodium diet	112 (79.4)
Help patients or clients understand that they should not stop taking their blood pressure medicine without talking to their doctors	104 (73.8)
Help patients or clients with keeping doctor’s appointments	103 (73.1)
Provide referrals to other social services	94 (66.7)
Educate about shopping for and preparing healthy foods	93 (66.0)
Help patients or clients understand they should talk to their doctors about any side effects they think their blood pressure medicines may have	93 (66.0)
Assist with goal setting	88 (62.4)
Offer or refer patients or clients to quit-smoking programs (smoking cessation)	80 (56.7)
Help patients or clients with insurance issues (eg, getting insurance, keeping insurance)	79 (56.0)
Help patients or clients with remembering to take medication by using pill boxes or other reminders	79 (56.0)
Help with transportation	76 (53.9)
Provide social support to patients or clients and family members	75 (53.2)
Provide blood pressure measurements	74 (52.5)
Counsel on filling and taking prescribed medicines as advised by doctors	73 (51.8)
Help people get free or low-cost blood pressure medicines	68 (48.2)
Provide in-home visits	66 (46.8)
Provide telephone or text appointment reminders	64 (45.4)
Assist with accessing exercise facilities	59 (41.8)
Offer translation services	54 (38.3)
Help people get free or low-cost home blood pressure monitors	52 (36.9)
Help with access to child care	29 (20.6)
Other^b^	6 (4.3)

a Respondents (who were aged ≥18 y, considered themselves to be community health workers, and spoke English) could check multiple items. Total does not equal 100%.

b Responses included give out cards with blood pressure readings to bring in to their next doctor appointment; check to see if they know how to use blood pressure monitors correctly; check if they have the right size cuff; advise how to manage daily stress situations; educate on how to track each measurement on an electronic device; teach how to use a medical tracking device; tell them to stop by for a blood pressure check if in the area; facilitate access to affordable primary care; and offer self-management programs.

Qualitative data indicated that CHWs accept patients’ understanding of hypertension and give their patients credit for what they already know. A CHW’s role in health education was not to provide basic information but rather to support patients in deepening their understanding of healthy lifestyles, reinforcing the doctor’s instructions, and providing support for the patient. One CHW stated, “Most of people know they shouldn’t be pouring salt on their canned beans already, but they still end up doing it because it’s there. It’s [important to] talk about the future and what’s going to happen. They can’t continue this if they want to live a healthy life” [Interview no. 2]. CHWs were invested in providing necessary health education material to their patients but viewed their role as intermediaries in the patient’s health education.

Another aspect of the CHW’s role in HSM was helping people receive necessary services. CHWs defined this role through their ability to connect patients with care through patient advocacy. Similarly, some CHWs discussed working in a clinical setting and had direct contact with the doctor, even sitting in on patient appointments to offer supplemental health education. CHWs also coordinated with doctors by connecting patients through referral programs.

In their hypertension management roles, CHWs used various delivery methods to work in partnership with patients (Table 2). The mean (SD) number of patients or clients with hypertension was 228.8 (715.5). Home visits averaged 39.7 (25.7) minutes, 7.8 (12.6) times per month over 7 (5.1) months. Identified clinical partners included nurses (49.7%), medical doctors (46.2%), health educators (31.7%), social workers (30.3%), and pharmacists (17.2%). When describing the partnership between CHWs and providers, CHWs reported that it was important to provide accurate information and to support the providers; they primarily reinforced HSM through the less clinical roles they were more familiar with (eg, hypertension-specific health education, providing referrals, advocacy).

**Table 2:** Hypertension Service Delivery Methods Used by Community Health Workers (n = 138^a^), United States, 2014

Method	No (%)
Individual in-person sessions in your organization’s setting (clinic, office)	85 (61.6)
Home visits	75 (54.3)
Outreach in community setting	66 (47.8)
Individual in-person sessions in community setting	62 (44.9)
Outreach in your organization’s setting (clinic, office)	58 (42.0)
Individual telephone and/or email sessions	56 (40.6)
Group classes or sessions in community setting	47 (34.1)
Group classes or sessions in your organization’s setting (clinic, office)	39 (28.3)
Other	1 (0.7)

a Number of participants who reported working with patients with high blood pressure.

### Medication adherence

Fewer than half (47.8%) of CHWs agreed or strongly agreed that their “patients are able to follow their high blood pressure medicine instructions.” CHWs contributed to reducing barriers to hypertension medication adherence through a patient-centered approach. The medication adherence work was not limited to high blood pressure medications but included navigating the intricacies of the health care system, reducing challenges with complex medication regimens, and alleviating fears through culturally appropriate methods. CHWs felt they were well equipped to address and support hypertension medication adherence according to WHO’s 5 dimensions of adherence (Table 3).

**Table 3:** Community Health Worker Responses Regarding Barriers to Medication Adherence and Roles of Community Health Workers in Medication Adherence for Hypertension, United States, 2014

Dimension and Description^a^	Barrier (% Reporting)	Role (% Reporting)
**Patient-related — **Inadequate knowledge and skills in managing the condition, lack of awareness about the cost and benefits of treatment. These barriers can be overcome through behavioral and motivational interventions, good patient–provider relationships, self-management, and memory aids and reminders.	N = 82	N = 84
	Forgetfulness of taking medicine (76%) Perceived consequences of not taking medicines (71%) Perceived need for treatment (65%) Forgetfulness of getting medicine refilled (61%) Perceived effectiveness of treatment (55%) Other (5%)	Counseling about changing health behaviors (eg, diet, physical activity, smoking) (92%) Counseling about the consequences of not taking medicine (88%) Counseling about necessity of treatment (81%) Counseling about the effectiveness of treatment (68%) Memory aids and reminders for taking medicines (63%) Memory aids and reminders for getting medicines refilled (52%) Other (2%)
**Condition-related — **Include lack of understanding about hypertension and poor perceptions about the disease. These barriers can be overcome through education on the use of medications.	N = 92	N = 84
	Multiple health issues (85%) Confusion if they are taking several medication for different medical conditions (83%) Understanding they need to stay on their blood pressure medicines (79%) Understanding of high risk of high blood pressure (76%) Keeping medicine refilled (65%) Taking correct dose of medicine at the right time (53%) Smoking (53%) Trouble reading medicine bottles (48%) Using or opening medicine bottles or containers (19%) Other (1%)	Encouraging healthy lifestyle (95%) Increasing knowledge about seriousness of high blood pressure (90%) Supporting patients with co-morbidities (86%) Encouraging smoking reduction or cessation (83%) Assisting with mental health and well-being issues (66%) Helping with medicine bottles (opening, closing, reading) (47%) Other (1%)
**Therapy-related — **Primarily include complex treatment regimens and adverse effects of treatment. To reduce these barriers, the World Health Organization recommends simplification of treatment regimens.	N = 81	Qualitative responses only
	Complex treatments (eg, patient takes more than 1 kind of medicine) (83%) Patients failure with taking medicines in the past (68%) Side effects (63%) — nausea, dizziness, fatigue Medicines change frequently (43%) Other (3%)	Education (following physician advice, medication education) Follow-up and monitoring Home visits Communicating with health care providers Communicating with pharmacist Reviewing changes in medicines Suggesting reminders, alerts, alarms, pill boxes, and labels
**Health system–related** **and health care team–related — **Lack of knowledge and training for health care providers on managing chronic diseases, poor patient–provider relationships, and lack of time by the provider. Training and education about medicines, positive patient–provider relationships, and continuous monitoring of self-management are interventions that reduce the health care barriers.	N = 92	N = 92
	Lack of knowledge by patient about high blood pressure medicine (82%) Inability to get medicine refilled (74%) Poor relationship between patient and doctor or provider (50%) Lack of communication among provider (49%) Access to health care provider (47%) Doctor or provider doesn’t know about patient needs (40%) Poor relationship between patient and pharmacist (37%) Other (7%)	Helping patients schedule appointments (85%) Helping patients access a health care provider (84%) Spending time with patients discussing health systems barriers (78%) Assistance with communication among providers (77%) Helping patients get their medicine refills (75%) Assistance with relationship between patient and provider (70%) Educating provider about patient needs (59%) Working with pharmacist (40%) Other (8%)
**Social and economic — **Illiteracy, unemployment, high cost of medicines, overall poor socioeconomic status. Interventions to address these barriers and improve adherence include family’s ability to help, patient health insurance, an uninterrupted supply of medicines, and sustainable financing for hypertension treatment.	N = 80	N = 80
	Cost of medicines (80%) Transportation (78%) Cost of care and/or insurance (75%) Literacy issues (69%) Social support (58%) Belief that medicines are a financial burden (56%) Other (6%)	Helping patients get low-cost medicine (81%) Helping patient’s family understand patient’s disease (77%) Helping patient get health insurance (75%) Helping with transportation needs (71%) Helping to read medicine label (69%) Other (7%)

a According to the World Health Organization’s 5 dimensions of adherence (23).

#### Patient-related factors

Three-quarters of CHWs reported people’s forgetfulness of taking medicine as a barrier to medication adherence. Additional patient-related barriers included perceived consequences of not taking medicines (71%), perceived need for treatment (65%), and forgetfulness about refilling medicines (61%). Over half of CHWs noted a barrier about attitudes about the perceived effectiveness of treatment. For example, one CHW described the lack of attention to taking medicines: “I think that one of the biggest problems is . . . realizing how important it is. Because it’s not a nagging heart attack, and those kinds of things don’t necessarily have continuous warning signs, there is a tendency to forget” [Interview no. 3]. CHWs described ways they assisted people with patient-related barriers through educational counseling (about health behaviors, consequences of not taking medicines) and encouraging medication adherence through memory aids for taking medicines and getting medicines refilled.

#### Condition-related factors

CHWs reported the most common condition-related barriers were having multiple health issues (85%) and confusion if they are taking several medicines for different medical conditions (83%). The most common health issues for this sample include diabetes (96.7%), obesity (88.9%), and depression or anxiety (87.8%). Over half of CHWs reported smoking as a condition-related barrier, but only 35.5% of CHWs agreed or strongly agreed that their patients or clients were trying to stop smoking. Eighty percent were actively encouraging smoking reduction or cessation. CHWs support condition-related barriers by encouraging healthy lifestyles (95%), increasing knowledge about the seriousness of high blood pressure (90%), supporting people with multiple conditions (86%), and encouraging smoking reduction (83%).

#### Therapy-related factors

Eighty-three percent of CHWs reported complex treatments as the most common therapy-related barrier. Other therapy-related barriers included failure to take medicines in the past (68%) and side effects (63%). The most common side effects reported were nausea (15%), dizziness (14%), and fatigue (10%). CHWs helped people who had complex treatments through education (eg, following physician advice, medication education), follow-up and monitoring, home visits, communication with the health care provider, communication with the pharmacist, going over changes in medicines, and suggesting reminders, pill boxes, and labels.

#### Health system and health care team factors

The most common barrier identified by CHWs was a lack of knowledge about hypertension medicines (82%), inability to get medicines refilled (74%), and poor patient relationship with their provider (50%). To address these barriers, CHWs assisted with scheduling appointments (85%) and accessing health care providers (84%). Approximately three-quarters of CHWs reported helping patients get the medicines refilled, assisting with communication among providers, and increasing patient’s access to their providers. CHWs support people with health system barriers by helping people navigate through the health system, facilitating communication with providers and pharmacists, and connecting people with the health care system.

#### Social and economic factors

The most common social and economic barriers included cost of medicines (80%), transportation (78%), and cost of care and/or insurance (75%). To address social and economic barriers, 81% of CHWs reported helping people get low-cost medicines. Over 75% of CHWs help people understand their disease and help with health insurance issues.

## Discussion

We sought to elucidate the roles CHWs perceive themselves playing in HSM and medication management with their community peers and health care providers. Our findings corroborate academic studies that report on CHWs’ effectiveness as interventionists for HSM control among underserved populations. Additionally, our study specifically identifies how CHWs drive successful patient outcomes.

CHWs offer an extension for HSM for the primary care provider. They interact with patients in meaningful and sometimes long-term relationships through individual clinical sessions or home visits. By extending the health care team into the community, CHWs are carrying out the chronic care model of care, a comprehensive, patient-centered approach to self-management (19). Its core elements are the community, the health system, self-management support, delivery system design, decision support, and clinical information systems. Our findings emphasize the role of the CHWs as influential for behavior change by linking self-management support in health systems and community settings. Providing truly team-based care allows for better communication and coordination of care among team members, improves team members’ use of evidence-based guidelines, provides a structured follow-up mechanism with patients, and helps engage patients in their own care (14). Engaging CHWs in team-based care to promote HSM activities improves blood pressure outcomes (eg, proportion of clients with blood pressure at goal) and cholesterol outcomes (eg, change in total cholesterol) (14). Earlier literature reveals positive patient outcome results. For example, 6 studies in the Community Guide’s comprehensive literature review demonstrated some improvement in medication adherence when CHWs worked with patients (14). Our study also highlights themes about barriers to medication management and how CHWs are helping to address barriers to improve adherence through offering memory aids and help with refilling prescriptions among other activities. CHWs are also a critical aspect of patient care coordination by facilitating navigation, referrals, and addressing their care plans. CHWs enhance provider communication and wellness behaviors, as recommended by self-management literature (20,21). Within a broader chronic disease–focused systems approach, CHWs could also reinforce critical self-management skills and competencies to address patients with multiple chronic diseases.

Our findings show that the CHWs interviewed are capable members of care teams for patients with hypertension. We suggest that research examine practical applications in health care and community settings to help CHWs maximize their skills and competencies to more effectively move patients to successful behavioral changes that will result in controlled blood pressure. Work may examine the link between the CHWs’ roles in chronic disease self-management and in policy and advocacy for patients, explicitly linking these features of CHW roles in their communities. Additionally, more information is needed about the dynamics of community pharmacists and CHWs’ partnerships to further explore opportunities in medication adherence interventions and improvement.

Opportunities exist for CHWs to work directly with community pharmacists to assist with health education at the point of medication pick-up, as patients may see their community pharmacists more frequently than their doctor. Thus, nurturing CHW–pharmacist collaborations may help improve medication adherence. Additionally, community organizations working with CHWs could provide training in cardiovascular disease prevention and self-management to increase CHWs’ comfort level (eg, knowledge) and skills and competencies (eg, accurate and approved method of blood pressure measurement, innovative self-management tools) (22). Organizations employing CHWs could partner with others, including state health departments and Area Health Agencies on Education, employers, and CHW networks to develop statewide standards for education and practice. These efforts would help CHWs, working in health care or other community settings, with patient documentation and assist with case management and record keeping. Further examination of how CHW interactions vary by population and best practices for scaling up the CHW roles and work (eg, delivery methods, increased patient load) are needed. CHWs are allies to providers in improving hypertension prevention and self-management, especially among underserved and diverse populations.

### Implications for providers

CHWs are well suited to be exceptional partners for providers hoping to improve their patients’ self-management skills or medication adherence or to expand their reach into community-based settings. To best work with CHWs, providers and systems should consider how to assess CHW performance, patient satisfaction, system integration or changes, and contributions they make to the prevention, treatment, and self-management of patients with hypertension and other chronic conditions. Clear guidelines paired with competency-based, ongoing training for CHWs and other team members are required to enhance and solidify their integration in the care team. Finally, novel, creative opportunities to include CHWs as partners in unique settings such as with community pharmacists should be considered, especially in challenging locations or among high-risk populations.

### Limitations

Limitations of this study include the exclusive focus on CHWs’ perspectives about HSM and medication adherence. CHWs are just one facet of the care team and their understanding of various barriers for self-management and medication adherence may be limited; it is important to consider the various team members involved in these efforts and how they influence the work of CHWs. In addition, CHWs from various recruitment channels self-selected to participate. Their reported activities and comments about their roles may not be representative of all CHWs. Additionally, WHO’s model for medication adherence is just one of many ways to measure and define adherence. This model aligns well with CHW roles but may oversimplify adherence issues. Finally, CHWs self-selected to participate; therefore, the sample may not be representative of CHWs nationwide.

### Conclusions

CHW interventions that activate patients to participate in their own care to improve their self-management and medication adherence behaviors are important for the prevention and treatment of heart disease and stroke. By understanding CHWs’ roles in HSM, health care professionals can better integrate CHWs into health care teams, thus enhancing opportunities to effectively address barriers for patients’ prevention and management of hypertension. CHWs can contribute to the reduction of hypertension disparities and health equity through their roles on health care teams. Ultimately, fine-tuned communication and collaboration among CHWs, other team members, patients, and community resources will improve population health equity and hypertension and cardiovascular disease outcomes.
